# Payment and economic evaluation of integrated care

**DOI:** 10.5334/ijic.2009

**Published:** 2015-04-22

**Authors:** Apostolos Tsiachristas

**Affiliations:** Health Economics Research Centre, Nuffield Department of Population Health, University of Oxford, Old Road Campus, OX3 7LF, Oxford, UK

Apostolos Tsiachristas, Doctoral Thesis

Erasmus University Rotterdam, The Netherlands, 2015, pp 253, ISBN: 978-94-6169-635-9

## Outline

Chronic diseases have an increasingly negative impact on (1) population health by increasing morbidity and mortality, (2) society by increasing health inequalities and burden to informal caregivers, and (3) economy by requiring enormous financial resources and jeopardising macro-economic development (e.g. consumption, capital accumulation, labour productivity and labour supply). Integrated care is the most promising concept in redesigning care to tackle the increasing threat of chronic diseases. Several European countries have experimented with models for integrating care, most frequently in the form of disease management programmes. These models were often supported by payment schemes to provide financial incentives to health care providers for implementing integrated care. This thesis aimed to investigate these payment schemes and assess their impact, explore the variability in costs of disease management programmes, and determine the costs and effects of disease management programmes.

## Introduction

A literature review and 15 interviews with experts in care for chronic diseases in Austria, France, England, the Netherlands and Germany were carried out to obtain detailed information regarding the payment schemes, facilitators and barriers to their implementation, and their perceived success. Focusing on the Netherlands, the most important conditions for the success of the new bundled payment scheme were identified based on literature, government documents, personal communications and site visits to disease management programmes. Furthermore, Organisation for Economic Co-operation and Development and World Health Organization data (1996-2013) were used to assess the impact of payment schemes that facilitate integration of care on health care expenditure across 25 European countries. Moreover, a prospective two-year observational study was performed to investigate the variability in costs and to assess the cost-effectiveness of 22 Dutch disease management programmes. In this study, data about quality of care, patient healthy behaviour and quality of life, and health care utilisation were collected via surveys and the development and implementation costs of disease management programmes were collected during interviews with disease management programme managers. Advanced regression methods (e.g. multi-level and repeated measurements analyses), sophisticated statistical techniques (multiple-imputation, propensity score matching, bootstrapping), subgroup and sensitivity analyses, and a thorough cost-utility analysis were performed to analyse the data. Furthermore, existing evaluation frameworks of disease management programmes were studied, practical field experience in the economic evaluation of disease management programmes was used and personal discussions with stakeholders in chronic care were carried out to develop a methodological framework for a comprehensive economic evaluation of disease management programmes based on multi-criteria decision analysis.

## Results and findings

The success of a payment scheme depends on the details of the specific implementation in a particular country, but a combination of the schemes may overcome the perverse incentives in each individual scheme. During a four-year period after implementation, pay-for-performance decreased the growth of hospital and administrative expenditure and all-inclusive agreements reduced the growth of outpatient expenditure. The results showed that financial agreements are potentially powerful tools to stimulate integrated care and influence health care expenditure.

Further, there is great variability in health care costs among patients included in disease management programmes which is explained by patient (e.g. multi-morbidity) and organisational (additional payment on top of the base payment for usual care) factors. There is also wide variation in development and implementation costs of disease management programmes, which is driven primarily by the duration of the development phase and the staff needed to develop and implement a disease management programme. These drivers are influenced by the attributes of the disease management programme, characteristics of the target population, project leadership and information and communication technology (ICT) involved. There are indications of economies of scale and economies of scope, which may reduce disease management programme development and implementation costs.

The results also showed that disease management programmes improve lifestyle indicators such as increased physical activity and reduced smoking in the short term. In addition, the results indicated that disease management programmes that are more comprehensive have the potential to be cost-saving, effective or cost-effective compared to less comprehensive disease management programmes.

## Implications for integrated care

A blended payment scheme was suggested to overcome potential barriers to the successful payment reform by providing an appropriate mix of financial incentives to health care providers and health payers. The structure of a health care system should be considered when designing such a blended payment scheme and the disease management programme development and implementation costs should be incorporated in the payment. Further, the pay-for-performance component should reward good performance and a shared savings scheme could be used to avoid ‘gaming’ and align the incentives of the stakeholders. Moreover, this thesis argued that finding the right mixture and density of disease management programme interventions that is optimally comprehensive for a specific target population and successfully implemented is the recipe to maximise the effectiveness of disease management programmes. The implications of the thesis’ findings were mainly related to the adequate definition and use of performance indicators, the design of a well-established ICT integrated system, the alignment of the disease management programme interventions with the targeted population, and the greater inclusion of health services in the bundled payment. This thesis calls for a comprehensive economic evaluation of disease management programmes that is not just based on a single criterion but takes into account multiple relevant criteria simultaneously. The framework developed in this thesis is a step towards standardising such an evaluation.

The results presented in this review are based on the author's thesis presented at Erasmus University Rotterdam on 20 March 2015.

## Published articles by the author relevant to this PhD thesis

Tsiachristas A, Cramm JM, Nieboer AP, Rutten-van Molken MP. Changes in costs and effects after the implementation of disease management programs in the Netherlands: variability and determinants. Cost Effectiveness and Resource Allocation 2014;12:17-7547-12-17. eCollection 2014.

Tsiachristas A, Waters B, Adams SA, Bal R, Molken M. Identifying and explaining the variability in development and implementation costs of disease management programs in the Netherlands. BMC Health Services Research 2014;14:518.

Tsiachristas A, Rutten-van Mölken MPMH. Exploring the variability of patient costs in disease management programs: a hierarchical modelling approach. Applied Economics 2014;46:940–951.

Tsiachristas A, Cramm JM, Nieboer A, Rutten-van Molken M. Broader economic evaluation of disease management programs using multi-criteria decision analysis. International Journal of Technology Assessment in Health Care 2013;29:301–8.

Tsiachristas A, Dikkers C, Boland MR, Rutten-van Molken MP. Exploring payment schemes used to promote integrated chronic care in Europe. Health Policy 2013;113:296–304.

Tsiachristas A, Hipple-Walters B, Lemmens KM, Nieboer AP, Rutten-van Molken MP. Towards integrated care for chronic conditions: Dutch policy developments to overcome the (financial) barriers. Health Policy 2011;101:122–32.

Boland M, Kruis A, Tsiachristas A, Assendelft W, Gusselkoo L, Blom C, et al. Cost-effectiveness of an integrated care program for COPD: The RECODE cluster randomised trial. European Respiratory Society 2014;Munich.

Boland MR, Tsiachristas A, Kruis AL, Chavannes NH, Rutten-van Molken MP. Are GOLD ABCD groups better associated with health status and costs than GOLD 1234 grades? A cross-sectional study. Primary Care Respiratory Journal 2014;23:30–37.

Cramm JM, Adams SA, Walters BH, Tsiachristas A, Bal R, Huijsman R, et al. The role of disease management programs in the health behavior of chronically ill patients. Patient Education and Counseling 2014;95:137–42.

Kruis AL, Boland MR, Assendelft WJ, Gussekloo J, Tsiachristas A, Stijnen T, et al. Effectiveness of integrated disease management for primary care chronic obstructive pulmonary disease patients: results of cluster randomised trial. BMJ 2014;349:g5392.

Boland MR, Tsiachristas A, Kruis AL, Chavannes NH, Rutten-van Molken MP. The health economic impact of disease management programs for COPD: a systematic literature review and meta-analysis. BMC Pulmonary Medicine 2013;13:40-2466-13-40.

Cramm JM, Tsiachristas A, Walters BH, Adams SA, Bal R, Huijsman R, et al. The management of cardiovascular disease in the Netherlands: analysis of different programmes. International Journal of Integrated Care 2013;Jul–Sep. Available from: URN:NBN:NL:UI:10-1-114736

Cramm JM, Strating MM, Tsiachristas A, Nieboer AP. Development and validation of a short version of the Assessment of Chronic Illness Care (ACIC) in Dutch disease management programs. Health and Quality of Life Outcomes 2011;9:49-7525-9-49.

